# Recent Trends and Developments in Conducting Polymer Nanocomposites for Multifunctional Applications

**DOI:** 10.3390/polym13172898

**Published:** 2021-08-28

**Authors:** Shubham Sharma, P. Sudhakara, Abdoulhdi A. Borhana Omran, Jujhar Singh, R. A. Ilyas

**Affiliations:** 1 Regional Centre for Extension and Development, CSIR-Central Leather Research Institute, Leather Complex, Kapurthala Road, Jalandhar 144021, Punjab, India; 2 PhD Research Scholar, IK Gujral Punjab Technical University, Jalandhar-Kapurthala, Highway, VPO, Ibban 144603, Punjab, India; 3 Department of Mechanical Engineering, College of Engineering, Universiti Tenaga Nasional, Jalan Ikram-Uniten, Kajang 43000, Selangor, Malaysia; 4 Department of Mechanical Engineering, College of Engineering Science & Technology, Sebha University, Sabha 00218, Libya; 5 Department of Mechanical Engineering, IK Gujral Punjab Technical University, Jalandhar-Kapurthala, Highway, VPO, Ibban 144603, Punjab, India; jujharsingh2085@gmail.com; 6 School of Chemical and Energy Engineering, Faculty of Engineering, Universiti Teknologi Malaysia, Johor Bahru 81310, Johor, Malaysia; ahmadilyas@utm.my; 7 Centre for Advanced Composite Materials, Universiti Teknologi Malaysia, Johor Bahru 81310, Johor, Malaysia

**Keywords:** biomedical, conducting polymers, corrosion, doped, electronics, shape memory polymers, sensors, actuators, optical limiting

## Abstract

Electrically-conducting polymers (CPs) were first developed as a revolutionary class of organic compounds that possess optical and electrical properties comparable to that of metals as well as inorganic semiconductors and display the commendable properties correlated with traditional polymers, like the ease of manufacture along with resilience in processing. Polymer nanocomposites are designed and manufactured to ensure excellent promising properties for anti-static (electrically conducting), anti-corrosion, actuators, sensors, shape memory alloys, biomedical, flexible electronics, solar cells, fuel cells, supercapacitors, LEDs, and adhesive applications with desired-appealing and cost-effective, functional surface coatings. The distinctive properties of nanocomposite materials involve significantly improved mechanical characteristics, barrier-properties, weight-reduction, and increased, long-lasting performance in terms of heat, wear, and scratch-resistant. Constraint in availability of power due to continuous depletion in the reservoirs of fossil fuels has affected the performance and functioning of electronic and energy storage appliances. For such reasons, efforts to modify the performance of such appliances are under way through blending design engineering with organic electronics. Unlike conventional inorganic semiconductors, organic electronic materials are developed from conducting polymers (CPs), dyes and charge transfer complexes. However, the conductive polymers are perhaps more bio-compatible rather than conventional metals or semi-conductive materials. Such characteristics make it more fascinating for bio-engineering investigators to conduct research on polymers possessing antistatic properties for various applications. An extensive overview of different techniques of synthesis and the applications of polymer bio-nanocomposites in various fields of sensors, actuators, shape memory polymers, flexible electronics, optical limiting, electrical properties (batteries, solar cells, fuel cells, supercapacitors, LEDs), corrosion-protection and biomedical application are well-summarized from the findings all across the world in more than 150 references, exclusively from the past four years. This paper also presents recent advancements in composites of rare-earth oxides based on conducting polymer composites. Across a variety of biological and medical applications, the fact that numerous tissues were receptive to electric fields and stimuli made CPs more enticing.

## 1. Introduction

**Figure 1:**
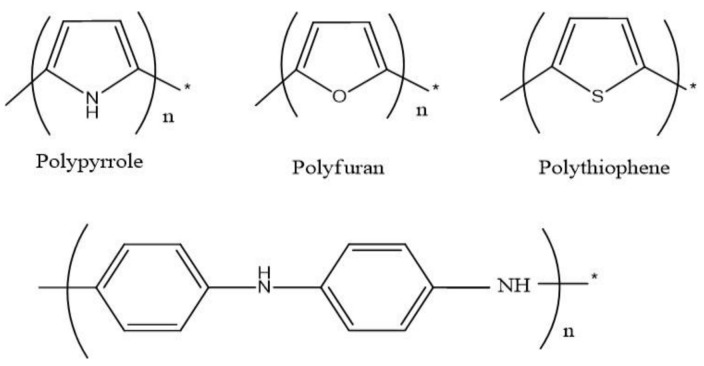
Intrinsically conducting polymers. Reproduced with permission from [1,2,3,4,5,6,7,8,9,10,11,12,13,14,15,16,17,18,19].

Approximately three decades ago, intrinsically conducting polymers were discovered and this discovery drew the attention of researchers because of countless applications of these polymers in the scientific field. These are also called synthetic metals as their electrical conductivity is very high, similar to those of metals. Examples of various conducting polymers (CPs) are polyacetylene, poly furan, polypyrrole, and polythiophene, which are insulators in their neutral state, as illustrated in Figure 1. The insulating behavior of polymers can be converted into conducting by carrying out doping of different salts by chemical and electrochemical redox reactions.

The highly conducting polysulfur nitride [SN]_x_ was discovered by Walatka et al. in 1973 [1]. MacDiarmid, Shirakawa, and Heeger enhanced the semiconducting behavior of organic polyacetylene in late 1970, which was synthesized by the chemical polymerization method. Their work on doping of polyacetylene with halogen derivatives was noticed and published in the chemical communication journal in 1977. These three scientists were conferred with the Nobel Prize in Chemistry in 2000 for discovering conducting polymers (CPs). After discovering conducting polyacetylene, scientists became interested in making other conducting polymers like polythiophene, polyaniline, polypyrrole, and polyfuran. Contrary to metals, these polymers can be processed at low temperatures, but their main problem is their stability. The conducting nature of these polymers is intrinsic due to their structure rather than by adding any conducting materials.

Fillers also play a pivotal role towards modification in the semiconducting and electrochemical performance of CPs. Since past decades, a wide range of chalcogenides derived from transition metals has been employed as fillers for modification in semiconducting [2] and electrochemical performance of CPs [3,20,21,22,23,24,25,26,27,28,29,30,31,32,33,34,35,36,37,38,39,40,41,42,43,44,45,46,47,48,49], as illustrated in Table 1. The product derived through the blending of such fillers with CPs is defined as PNCs. The electrochemical supercapacitance of such PNCs is well documented [50,51,52,53,54,55,56,57,58,59,60,61,62,63,64,65,66,67,68,69,70,71,72,73,74,75,76,77,78], whereas limited research has been made on the implication of rare earth oxides (REOs) as fillers for semiconducting and electrochemical applications, as demonstrated in Table 1 [79,80,81,82,83,84,85,86,87,88,89,90,91,92,93,94,95,96,97,98,99,100,101,102,103,104,105,106,107,108,109,110].

The electrical and electrochemical characteristics of REOs vary in a size dependent manner. Furthermore, PNCs derived through blending REOs with CPs offer a wide spectrum of applications such as solid polymer electrolytes [4], semiconductors [2], windows in dye-sensitized cells [5], electrochemical behavior, and charge storage [6,7,8]. Common CPs involved in synthesis of REOs based PNCs are PPY [9], PTh [10], PANI [11], and PIN [12].

**Figure 2:**
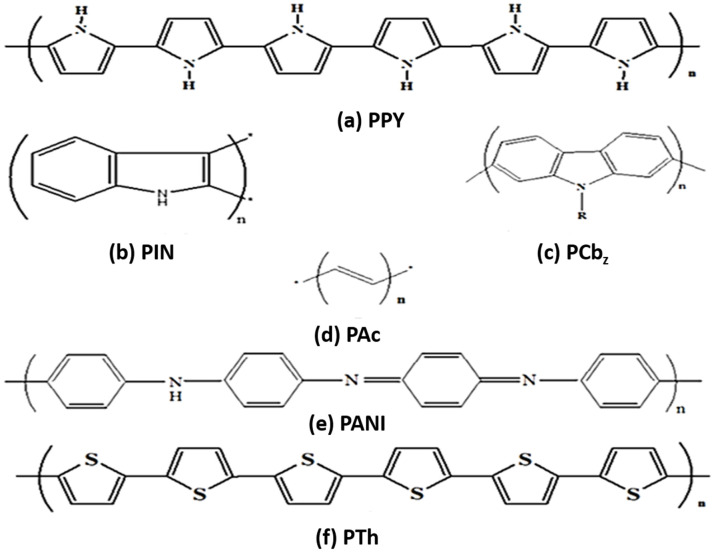
(**a**). PPY; (**b**). PIN; (**c**). PCbz; (**d**). Pac; (**e**). PANI; and (**f**). PTh-based CPs are used for semiconducting and electrochemical applications. Reproduced with permission from [9,10,11,12,13,14,15,16].

The electrical conductivity of electrodes is routinely recorded at variable temperatures without taking the cognizance variations in their microstructure. The electronic and electrochemical significance of CPs has been well documented since decades [11,13,14,15]. CPs commonly utilized for charging a storage battery and semi-conducting applications are PPY, PIN, PCbz, PAc, PANI, and PTh [16], as illustrated in Figure 2a–f.

In 1987, Heeger and his coworkers used polythiophene for making diodes for electronic devices applications and then developed high efficiency polymer-based LEDs. These polymer LEDs have been used to make emission displays, which were used in cell phones in 2003 [17].

The various applications of conducting polymers can be increased by doping with other functional materials to form polymer composites [18]. These are used in different fields like physics, chemistry, electronics, and biomedical science [19].

**Figure 3:**
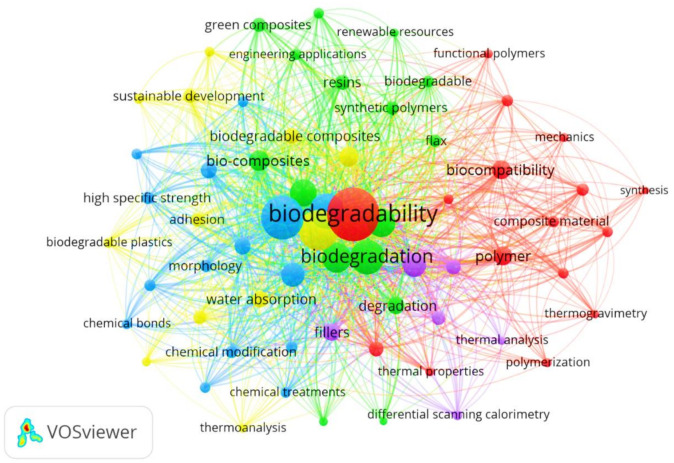
Systematic mapping summary of scientific advancements on polymer bio-nanocomposites for Multifunctional applications in anti-static, anti-corrosion, actuators, sensors, shape memory alloys, biomedical application, flexible electronics, solar cells, fuel cells, supercapacitors, LEDs, and adhesive domains.

Conducting polymers containing metal particles possess interesting properties of scientific and practical interests [20]. During the past few decades, researchers are paying more attention in conducting polymer composites to develop some new properties that were not observed in individual materials [21,22]. Researchers are more interested in developing the three-dimensional structure of conducting polymers, hybrid and nano hybrid materials of conducting polymers. The hybrid and nanohybrid conducting polymers are synthesized by adding metal, metal oxides, graphene, graphene oxide in conducting polymers. These new materials improve functionality in different areas like sensors, electronic devices, and biomedical application. The graphene nano hybrid of these polymers is used as an electrode in synthesis of capacitors. These nanohybrid materials increase stability, flexibility, and capacitances of capacitors [23]. Such polymers can be deposited either chemically or electrochemically on the metal. The different properties of polymers, like thermal stability, mechanical properties, conductivity, and corrosion protection properties on steel and aluminium, can be improved by doping. The doped conducting polymers have more capability for corrosion protection than undoped polymers because they give a suitable environment for corrosion protection on metal surfaces by restricting movements of corrosive agents or forming a uniform passive layer of doped polymers on metal surfaces [24,25,26]. Figure 3 reveals the bio-informatic visualization, which exhibits current progressions on the polymeric nanocomposites in a broader spectrum of anti-static, anti-corrosion, actuators, sensors, shape memory alloys, biomedical application, flexible electronics, solar cells, fuel cells, supercapacitors, LEDs, and adhesive domains using the Vosviewer Scientometric analysis.

## 2. Synthesis of Conducting Polymers

In the available literature, different ways to produce inductively coupled plasma (ICP) have been demonstrated. The polymerization process forms a solution containing the monomer is either a chemical or electrochemical process.

### 2.1. Chemical Polymerization

**Figure 4:**
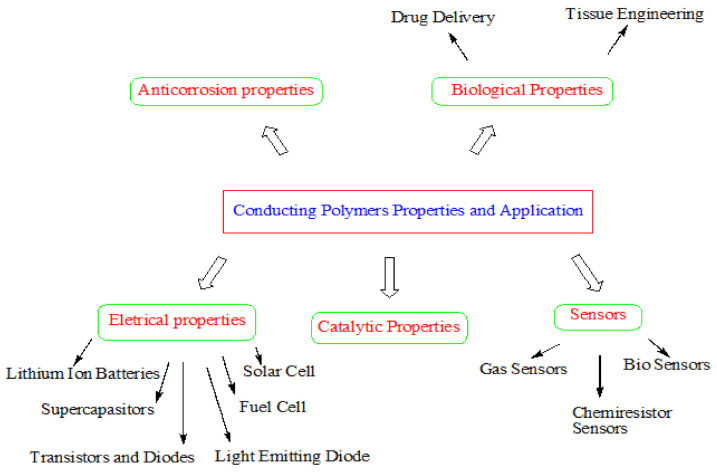
Reaction showing Chemical Polymerization of aniline. Reproduced with permission from [27,28,29,30].

In this polymerization, monomers can be polymerized by various oxidizing agents like ammonium per sulphate, hydrogen per oxides, etc. [27]. The chemical polymerization of aniline is shown in Figure 4. This type of polymerization occurs by any of the methods: Addition polymerization and step growth polymerization. An oxidant is used to polymerize the monomer, and anions are doped as a counter part of the oxidative CP. This method to produce ICPs is widely used in industry.

Polyaniline and polypyrrole were synthesized on various substrates such as Pt, Au, Fe, Al, stainless steel, carbon fibers, brass, and zinc [28,29,30]. Isomers of poly-toluidine have been synthesized using the chemical oxidation method at 0 °C using potassium dichromate as oxidant and hydrochloric acid as dopant [31]. Polyaniline composites doped with TiO_2_ were also synthesized by this method [32]. Polypyrrole doped with various dopants like Lithium per Chlorate (LiClO_4_), para-Toluene Sulfonate (p-TS), and Naphthalene Sulfonic acid (NSA) was synthesized by chemical polymerization [33]. Polyaniline doped with tungstate was also chemically synthesized and characterized by various techniques [34].

The composite films of polypyrrole and polyvinylidine fluoride were formed by the chemical oxidation method and ammonium per sulphate used as an oxidant [35]. The nanocomposites of polypyrrole with copper sulfide were synthesized and characterized by various techniques [36].

Polymerization of furan by acidic catalysts has been reported by various researchers [37,38]. Armour et al. observed the electrical conductivity of polyfuran, which was synthesized chemically utilizing trichloroacetic acid [39]. Polyfuran was synthesized using pyridinium chlorochromate (PCC) as oxidizing agent [40].

Pyrrole was polymerized by chemical an oxidation method in the presence of Fe_2_(SO_4_)_3_ and surfactant. The surfactant and oxidizing agent increased the conductivity and yield of polypyrrole [41]. Polypyrrole doped with tungstate or vanadate was synthesized by the chemical polymerization method and characterized by various techniques [42].

### 2.2. Electrochemical Synthesis

Electrochemical methods also synthesize the conducting polymers. It is very simple and a better technique for the preparation of conducting polymers because, in this technique, polymerization and doping levels could be controlled [43]. In this technique, three electrodes, working, counter, and reference electrodes, are required.

The physical properties of CPs coating are affected by the nature and size of counter ions used. The properties of conducting polymers like thermal and mechanical can be improved by the incorporating sulfonated aromatic ions [44]. The coating of poly (N-methyl pyrrole) doped with TiO_2_ deposited on steel substrates by this method was studied [45]. PPy/TiO_2_ nanocomposites were synthesized, and these composites are used for paint application [46]. The electro-polymerization of polyaniline, polypyrrole, and their composites was carried out on stainless steel and aluminium using the cyclic voltammetry technique [47,48]. Oxalic acid and tungstate doped polypyrrole films were potentio-statically electro-polymerized on the surface of aluminum alloy 1100 [49]. Polyaniline composites doped with tungstate and molybdate were synthesized by the electrochemical method [50,51]. Polypyrrole composites doped with zinc phosphate were deposited on AISI 1010 steel [52]. The copper doped polypyrrole was synthesized on steel by an electrochemical method for corrosion protection [53]. Table 2 exhibits the comparison of chemical and electrochemical polymerization synthesis methods with their respective merits and demerits.

**Figure 5:**
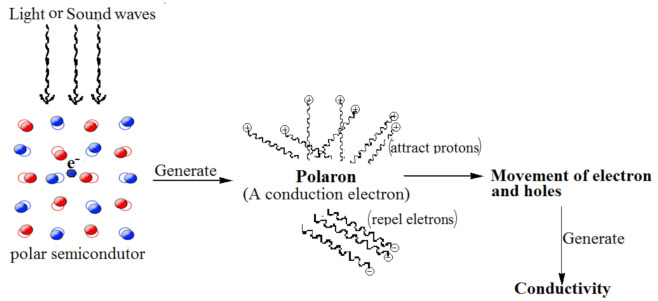
Conduction mechanism through polaron theory.

The semiconducting and electrochemical performance of PNCs is evaluated as to their working electrode (WE) fabricated through coating composition of carbonaceous material with CPs and a polymeric binder. Common carbonaceous materials employed for the fabrication of WE are graphite and its tubular nanostructures. CPs used are either synthesized or commercially procured. The common conduction mechanism in rare earth oxides (REOs) (polar) has been explained through polaron theory, as revealed in Figure 5.

Interaction involving conformation degree of freedom through semi-conducting polymerics could localize an electric charge, and also have a substantial impact on carrier mobility, energy, and mass-transfer. Polarons could arise whenever charged particles cause aberrations, distortions, and abnormalities in the surrounding media, like locally stretching vibratory patterns or dielectric polarization. Such deformations, subsequently, come into contact with the charge-particles in an appealing manner, striving to localise it. First, the development of vibrating polarons in conducting polymers using a firmly binding model paradigm for charge hops across adjoining rings, which is linked to ring deformities. The coupling constants employing theoretical calculations, and molecular dynamics, for ring aberrations, and the couplings to the charged particles and carrier mobility. One such mechanism produces mainly wide, loosely knit/linked polarons on individual chain-rings. Furthermore, the polarons stabilized via dielectric polarization, which have been semi-classically characterized by a charged polarizable continua, are then interacting with the charge-carrier wave-function. Di-electrically stabilised polarons, in contrary to vibrating polarons, are narrower, more firmly coupled, and persistent, and stabilized in two-dimensional crystallographic layers.

An excitation activating energy level indicates the presence of a relaxed mechanism (a conduction activity), which can be characterized as polaron-hopping among adjacent locations in within the crystalline lattice structure. Through use of lattice oscillating, it is necessary to mobilise the confined localized electron. Among these instances, electrons are assumed to travel through hopping movement stimulated by lattice crystalline oscillating, i.e., a conducting method is supposed to be multiphonon-assisted hopping of tiny polarons across localized regions.

**Figure 6:**
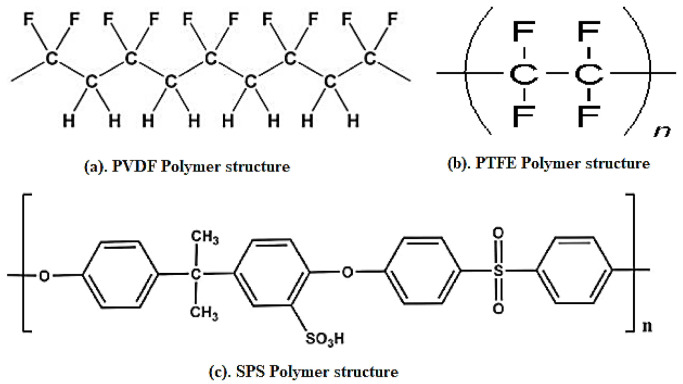
Binders, (**a**). PVDF-polymeric structure; (**b**). PTFE-polymeric structure; and (**c**). SPS-polymeric structures, used for electrode fabrication. Reproduced with permission from [11,13,14].

Doping with acidic functionalities reduces the band gap that enhances the conductivity of PPY [54]. Semiconducting components and electrodes for charge storage devices are routinely developed through either of chemical or physical vapor deposition over semiconducting wafers. Alternatively, PPY electrodes are produced through electrode position over metals or by hand laying of a composition of PPY over metallic substrates in presence of graphite, dopants and polymeric binders [11,13,14]. Common polymeric binders employed for the development of WE are polyvinylidene fluoride (PVDF), polytetrafluoroethylene (PTFE), and sulphonated polysulphone (SPS), as shown in Figure 6.

## 3. Properties and Multifunctional Applications of Conducting Polymers

**Figure 7:**
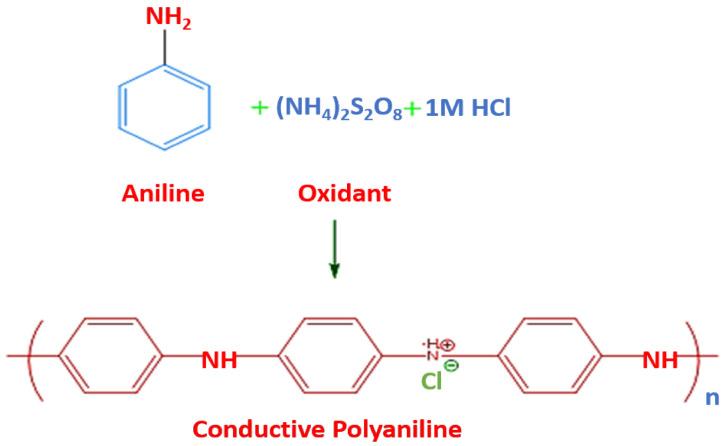
Numerous applications and properties of conducting polymers. Reproduced with permission from [62,63].

Conducting polymers have wide applications in various fields such as supercapacitors, electrochromic devices, biosensors, and electrocatalysts [55,56,57]. Conducting polymer nanocomposites of inorganic oxides have various applications in the field of chemistry and physics due to their electro-optical properties [58]. Various applications and properties of conducting polymers are shown in Figure 7. Several properties of conducting polymers like processability, conductivity, permeability, and mechanical properties are increased by dopant anions [59,60,61].

### 3.1. Electrical Properties

Conducting polymers have various applications in electronic devices like batteries, solar cells, fuel cells, and supercapacitors due to the highly conducting nature of polymers. Various applications of CPs are described below.

#### 3.1.1. Lithium-Ion Batteries

Conducting polymers have been used in batteries. Several polymers like polypyrrole, polyaniline, and polyacetylene are used as electrodes in batteries. PPy composites doped with MnCo_2_O_4_ are used as anode in lithium-ion batteries. These composites have good stability, a high-performance rate, and are light weight [62]. These batteries are used in electrical vehicles, mobile phone, and tablets.

Since lithium-ion batteries have such a complicated intricate architecture framework, all electrode elements, comprising active materials, binding additives, conductive agents, electrolytes, and membranes, must be designed in an inventive manner. One such review demonstrated how physicomechanical deterioration might have been whittled down by replacing the conventional PVDF/C electrodes matrices with CP-based bindings as elastic and adherent conducting materials. This is one way whereby research towards passive components and composite materials extends the electrode surface, active material, absorber layer area through shifting prerequisites and demands for active materials, like lower volumetric changes, to passive components, which could handle those demands, like flexibility, adhesion-conducting [62].

Multi-functionalized CP-based binding adhesives have demonstrated intriguing characteristics like suitability with solvent-treatment, appropriate electrochemical-ionic, carrier-mobility properties, significant adherence to active components, and probable capacities contributions. Through formation of composites with nonpolar hydrophile polymerics, altering CP side-branches with hydrophilic groups, and manufacturing a hydrogel-derived three-dimensional electrode structure of conducting polymeric active components through in-situ-polymerization, investigators have already also addressed the inherent and profoundly poor processing of pristine CPs. The previous two methods are appealing due to their ease of synthesis and scaling reproducibility at an affordable price. Another technique inherited the benefits of hydrogel materials, like adjustable nanostructures and centralized hierarchical three-dimensional electrode structures comprising interconnecting ionic and electronic conductions. Furthermore, consequentially, the above-mentioned designing notion for CP-based binding adhesives, specifically hydrogel-based CP composites, might discover usage in energy storage devices, rechargeable-batteries, super-capacitors, and flexible/elastic equipments [62].

#### 3.1.2. Solar Cells

Conducting polymers have been used in solar cells. These are used as an electro catalyst in solar cells. PPy aluminium oxide composites were used as electro catalyst for solar cells. Dye sensitized and photovoltaic solar cells based on conducting polymers are used in the place of silicon solar cells because these have high energy conversion efficiency and a lower cost than silicon-based solar cells. These are also used as energy transfer mediators in solar cells [63].

Conducting polymers have found widespread application in solar-sectors like dye sensitized solar cells (D.S.S.C.), perovskite-structured solar cells (P.S.C.), and organic/polymer-photovoltaics (O.P.V.).

Polymers could be utilised in D.S.S.C. as not only an elastic adjustable surface, but rather as pore-forming and films-forming agents of photo-anode-films; likewise, owing to their rising catalytic-properties, conductive polymerics as well as comparative composite materials are being employed to manufacture Pt-free counter-electrode materials; and in PSC, polymers could indeed be utilized to promote nucleating polymerization, crystallization, and control crystal-growth. Because of its remarkable energy-gap and charge carrier mobility, polymerics could also be employed as hole-transporting materials. Developing innovative polymeric hole-transporting materials with higher charge carrier mobility and optimal binding energy-level design layout is problematic [63].

Conducting polymerics are extensively employed as active absorber layered-structure in O.P.V.s to affect photo-harvesting efficiencies and equipment operating performance parameters. The technique of obtaining escalating efficiency O.P.V.s seems to be the emergence of various polymeric donors/drivers with a narrower band-gap as well as optimal energy barrier frameworks [63].

#### 3.1.3. Fuel Cells

In the past few decades, fuel cells had various advantages for applications in electric vehicles, such as automobiles [64]. Polymer fuel cells are of two types: low temperature and high temperature fuel cells. The membrane of high temperature fuel cells is made with poly (benzimidazoles). Direct methanol fuel cells (DMFCs) have been used in the field of energy applications because they have fuel portability and high energy conversion efficiency [65]. Conducting polymers with 1D-nanostructures are used as electro catalyst supports in cells [66].

Even though conducting polymeric materials have distinctive characteristics such as rising electron-carrier charge mobility, conductivity, and electrochemical performance, owing to the existence of the organic conjugated polymeric backbone, excellent electron de-localization from of the CPs to the hybrid material, and higher surface-area, such characteristics assist in making conducting polymeric-based nano-hybrids (C.P.N.H.s) an appealing material for analyzing their physical and chemical characteristics but also continuing to expand their application areas (P.E.M.F.C.s) to various sorts of polymeric electrolytic-membrane fuel cells. Till date, relatively few polymer multimetallic effectual electro-catalyst-supported CPs have been explored, and transitions material-based C.P.N.H.s have also still not been disclosed. These steep prices have had the ability to strengthen the catalysis performances and functionality and efficiency of fuel cell energy storage equipment [65,66].

#### 3.1.4. Light Emitting Diodes (LEDs)

Conducting polymers like Poly(p-phenylenevinylenes) (PPVs), Poly(dialkylfluorenes) (PFs), Polythiophenes (PThs), and their derivatives exhibit potential for polymer light-emitting diodes (PLED) applications [67,68]. By introducing bulky phenyl side groups in the polymer, the performance of PLED could be improved [69,70].

#### 3.1.5. Supercapacitors

**Figure 8:**
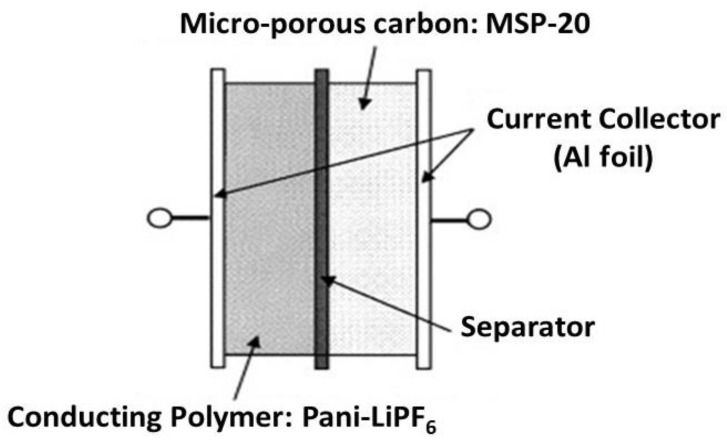
Hybrid type supercapacitor. Reproduced with permission from [75].

These are energy storage devices and are used in solar arrays, hybrid electric vehicles. They have intermediate specific energy between batteries and capacitors. They have a high charging and discharging capability. The supercapacitors based on conducting polymers have high charge storage efficiency so they can store a large amount of energy [71]. Conducting polymers have been used as active electrode materials for supercapacitors due to high conductivity, flexibility, stability, and low cost [72,73,74]. The hybrid type supercapacitor is shown in Figure 8.

### 3.2. Anticorrosion Properties

These days, conducting polymers and their composites have been used as corrosion protecting agents on metal surfaces [76,77]. Corrosion protecting behavior of these composites is due to the capacity of inhibit the movement of corrosion causing agents on surface of metals [78,79,80]. It was studied that the polyaniline coating on the steel surface protects from corrosion by forming the passive film [81]. Polyaniline epoxy blended coating on steel as a corrosion inhibitor has been studied [82]. Polyaniline/polypyrrole and polyaniline-polypyrrole phosphotungstate composites were used as corrosion protecting agents on the mild steel surface. Composite films give better corrosion protection than bare polyaniline and polypyrrole [83]. Polyaniline doped with TiO_2_ nanoparticles (PTC) were used as corrosion protectors, and they were more effective than undoped polyaniline [32].

Oxalate, as well as tungstate doped-PPy, used as a corrosion protector on aluminum has been observed [49]. It was observed that the PANI-MoO_4_^2−^ coating acts as a better corrosion inhibitor as compared to pure PANI coating [51]. Polyaniline and its composites films possess corrosion inhibition properties [84,85]. It was studied that zinc phosphate-doped PPy gives better corrosion protection than undoped PPy [52,53]. Poly-6-amino-m-cresol doped with copper nanocomposites give corrosion protection of mild steel. These composites give better performance than bare polymers [86]. The corrosion behavior of 7075 aluminum, copper modified Al, polypyrrole modified Al, and copper/polypyrrole modified Al samples were noticed.

### 3.3. Catalytic Properties

Conducting polymers have been used as electrocatalysts and photocatalysts in biosensors, cells, and energy-related devices because of high conductivity and electroactive properties of conducting polymers. The high conductivity of conducting polymers increases the efficiency of charge transfer between electrode and electrolyte, which improves catalytic activity. These are used as a catalyst for enzymes in electrochemical sensors. The nanocomposites of polymers were used as photocatalysts [87]. The nanocomposites of polypyrrole-titanium dioxide showed more photocatalytic activities in the degradation of Rhodamine B than pure TiO_2_ [88]. Fe_3_O_4_/Pd@PPy composites showed superior catalytic activity and better stability in successive cycling tests [89].

### 3.4. Sensors

In the recent past, polymer nanocomposites have emerged as the most promising materials for cost effective sensors with excellent sensitivity and selectivity with fast and reliable sensing techniques. The unique electrical, thermal, and optical properties of graphene when combined with the light weight, good processability, and excellent mechanical properties of polymers, offer a new class of materials capable of fulfilling the stringent requirements for a wide variety of sensors. Graphene–polymer nanocomposites have attracted enormous interest, mainly due to the fact that exceptionally high performing composites can be prepared by the use of extremely small quantities of the filler due to their nano-level dispersion in the polymer matrix. Basically, ‘Sensors’ detect the changes in any of its physical properties and convert them into a measurable signal. Based on the changes in their optical, electrical, chemical, electrochemical, and mechanical properties, graphene–polymer nanocomposites can be used as biosensors, chemical sensors, gas/vapor sensors, strain sensors, etc.

Conducting polymers have wide applications in sensors like gas sensors, bio sensors, optical sensors, strain sensors, and chemiresistor sensors.

#### 3.4.1. Gas Sensors

Conducting polymers are used in gas sensors. These are employed in forming the active layer in sensors due to the conductive and flexible nature of conducting polymers. Gas sensors have a wide range of applications in different areas, like industrial production, food processing, environmental monitoring, and health care, etc. [90,91,92]. Conducting polymers and doped conducting polymers with different metal salts have been used in gas sensors. Polypyrrole film with various dopants has been used in gas sensors [93,94].

Biochemical sensors are made of a responsive component towards a specified analyte (molecular-identification) and transduction processor, which converts analyte-concentrations into measurable input-physical electrical signals like voltage, absorption, current, or mass. Gas sensor devices based on CPs were being categorized by IUPAC dependent on input-signaling waveform transduction. Sensors appertaining to the chemically modulating of electrochemical, optoelectronic, or physicomechanical transmission and propagation processes of CPs would be thoroughly addressed through consideration with gas-sensing applications.

Around ambient temperature, the contact interface among the conducting polymer as well as the gas analyte is still quite significant. As a result, sensors predicated on conducting polymerics could provide spectacular signal output responses, whereas those dependent on synthetic transition metal-oxide have almost zero responsiveness at room temperature. Consequentially, conducting polymeric sensing devices require less energy and also have a simplified device architecture.

#### 3.4.2. Bio Sensors

Conducting polymers are very useful for the expansion of biosensors because these are good materials for the immobilization of biomolecules. Conducting polymers and their composites are used in fabrication of different biosensors and also improve speed and sensitivity of biosensors. Delocalization of electrons in conducting polymers is very fast, which is good for efficient biosensors [95]. The conducting polymers provide suitable environment for immobilization of enzymes and biomolecules on the electrode surface. The enzymes and biomolecules may be amalgamated in conducting polymer films during electrochemical deposition on electrodes. The amalgamation of enzymes in conducting polymers gives proximity between active site of the enzyme and conducting surface of the electrode, making it suitable for biosensor construction. Glucose oxidase can be successfully amalgamated in polypyrrole films for glucose detection [96]. The biosensors based on conducting polymers were discovered to treat penicillin and detect innumerable chromosomal disorders. A highly sensitive and rapid flow injection system for urea analysis was fabricated using the composite film of polypyrrole and a polyion complex [97]. Glucose biosensors are used to estimate glucose by the arrest of glucose oxidase enzymes with conducting polymers. The DNA biosensors based on conducting polymers have been investigated for diagnosing and treating of various diseases like chromosomal disorder by repairing, degradation, or multiplication. Biosensors have a great role in environmental monitoring by controlling various hazardous chemicals like formaldehyde and hydrogen peroxide, which cause pollution in the environment [98].

#### 3.4.3. Chemiresistor Sensors

The conducting polymers have also been used in chemiresistor sensors due to their conductivity. The conducting polymers play an important role in sensors because they can act as both electron donors and electron acceptors when interacting with the gaseous form. The conductivity of conducting polymers increases when it acts as an electron donor to gas and decreases when it acts as an electron acceptor to gas [99]. Pt, Au, and Pt-Ni IDAs pre-patterned over alumina, quartz, glass, acrylic strip, silicon chips, Si_3_N_4_/Si are normally used as chemiresistor sensors [100].

#### 3.4.4. Strain Sensors

Strain sensors measure a local deformation due to an applied strain. Strain sensors mainly find application in damage detection, structural health monitoring, and structural and fatigue studies of materials. The outstanding electrical properties of graphene have made it the most promising material for developing strain sensors. In general, graphene-based strain sensors can work mainly based on three mechanisms:

- based on structure deformation of graphene,
- based on over connected graphene sheets and finally,
- based on tunneling effect of neighboring graphene sheets.

The structural deformation of graphene due to an applied strain can induce attractive changes to its electronic band structure and electrical properties. There have been a lot of theoretical calculations to find out the effect of different types of strain on graphene. Experimentally, strain sensors based on the structural deformation of graphene have already been demonstrated by many researchers. CVD-grown graphene, transferred on flexible polydimethylsiloxane (PDMS) substrate, showed a gauge factor of ~151 [101]. When the strain was applied, initially, the resistance was decreased, which was due to the relaxation of pre-existing wrinkles on the graphene sheet.

Further increase in strain resulted in increase in resistance due to distortion of the hexagonal honeycomb crystal structure of graphene. Even though these sensors have high sensitivity, the large strains can cause unrecoverable structural deformation, limiting their practical application. Researchers made efforts to overcome this limitation by forming rippled graphene layers on pre-strained PDMS [102]. The graphene layer was deposited on a pre-strained PDMS, and when the strain of the PDMS was released, the graphene layer above the PDMS formed a rippled structure. The resistance of this sensor was found to linearly decrease with increasing strain. The resistance changed from 5.9 kΩ to 3.6 kΩ when the strain was increased from 0% to 20% till the graphene layer was completely flat.

### 3.5. Actuators

These features make them the most promising candidates for many applications, including artificial muscles and robotics. Graphene polymer composite actuators are considered one of the most versatile actuator systems.

The electrically triggered polymer actuators can follow two different mechanisms: either utilizing Maxwell’s stress, which is generated as a result of the electrostatic attraction between the two electrodes, or by pure electrostrictive effect [103]. However, there can be another class of polymer actuators in which the large mechanical deformation is caused by ionic charge movement at lower voltages like in ionic polymer based metallic composites [104]. During the last few years, there have been several reports to develop electromechanical actuators with excellent actuator performance by combining the unique properties of graphene with different polymer systems. Unlike the conventional polymer based electromechanical actuators, graphene–polymer composite actuators follow different mechanisms in different systems depending on the materials and the design of the actuators. This makes them the most versatile actuator materials for various practical applications.

**Figure 9:**
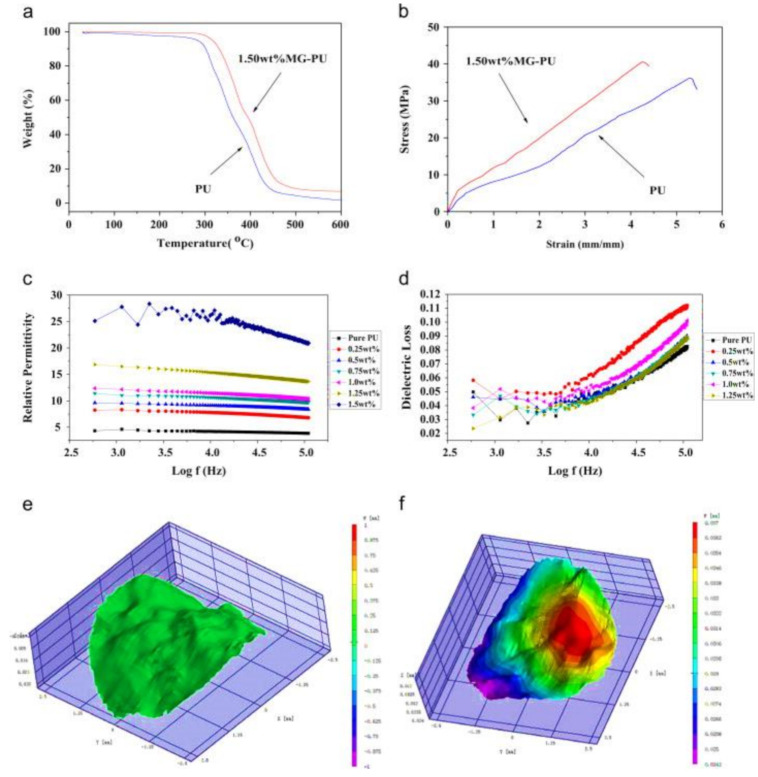
DTA curve (**a**). Load-deflection curves; (**b**) of Polyurethane and 1.5 wt. percent poly(methylmethacrylate) functionalized graphene–polyurethane, dielectric-constant; (**c**) and dielectric-loss; (**d**) of poly(methylmethacrylate) functionalized graphene–polyurethane composites, electrical field induced strain-nephograms; ((**e**) Voltage-off, (**f**) Voltage-on) of 1.50 wt. percent poly(methylmethacrylate) functionalized graphene–polyurethane composite film. Reproduced with permission from [105].

In a study by Chen et al. using poly(methylmethacrylate) functionalized graphene–polyurethane (MG-PU) composite actuators, it was found that the introduction of functionalized graphene into the PU matrix significantly improved the electric field induced strain behavior when compared to pure PU films. The electrical field-induced straintronics increased from 17.6 per cent for pure PU to around 32.8 per cent for 1.5 wt. per cent, MG-PU composite film and was nearly two-timing that of pure film as presented in Figure 9 [105].

**Figure 10:**
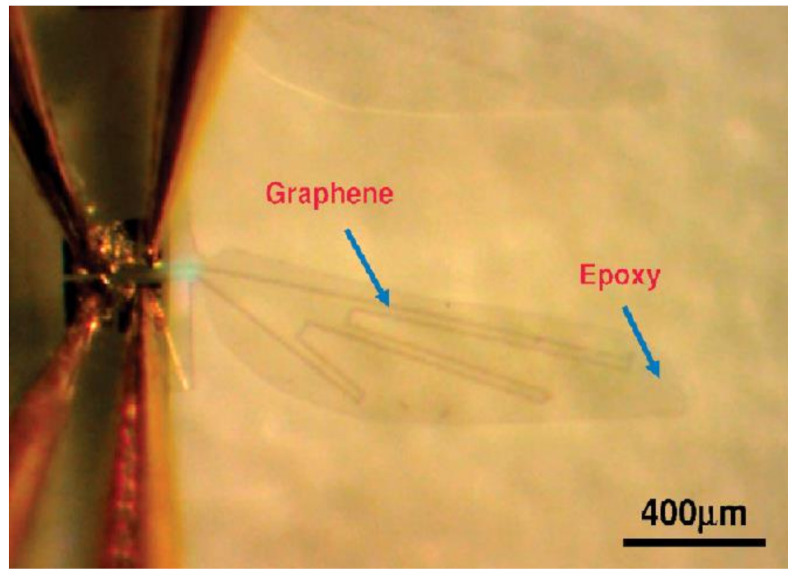
A Graphene on organic film in the form of a dragonfly wing. Reproduced with permission from [106].

Graphene can undergo contraction on heating due to its negative temperature coefficient of thermal expansion. This unique property was utilized by Zhu et al. for designing a bimorph actuator using a graphene–epoxy hybrid system [106]. The graphene-on-organic film actuator was developed as a cantilever in which graphene acted both as the conducting layer and heating layer. Upon applying electric power, the graphene was directly heated, and the epoxy was warmed up by diffused heating. Due to the mismatch in thermal expansion of graphene and epoxy, the cantilever exhibited a deflection towards the graphene coated side. The device exhibited high actuation behavior at very low power. For instance, the cantilever tip deflected 1 µm with an input voltage as low as 1 V within 0.02 s and returned back to its original position within 0.1 s. They have also reported that the flapping and bending motion of the actuators can be controlled by changing the frequency and duration of applied voltage. They have demonstrated the working of this graphene on organic film actuator in the form of a dragon fly wing, as illustrated in Figure 10.

A similar bimorph actuator based on a bilayer of graphene and polydiacetylene (PDA) was reported by Liang et al. [107]. The actuator generated a large actuation motion under a low electric current in response to both dc and ac signals. For example, for a bimorph of size 10 mm length by 2.7 mm width, at a very low dc current of 20 mA, a displacement as large as 1.8 mm and curvature of 0.37 cm^−1^ were obtained. Similarly, at applied DC of just 0.29 A/mm^2^, actuation stress as high as 160 MPa/g was obtained. Under the ac signal, this actuator displayed reversible swing behavior.

**Figure 11:**
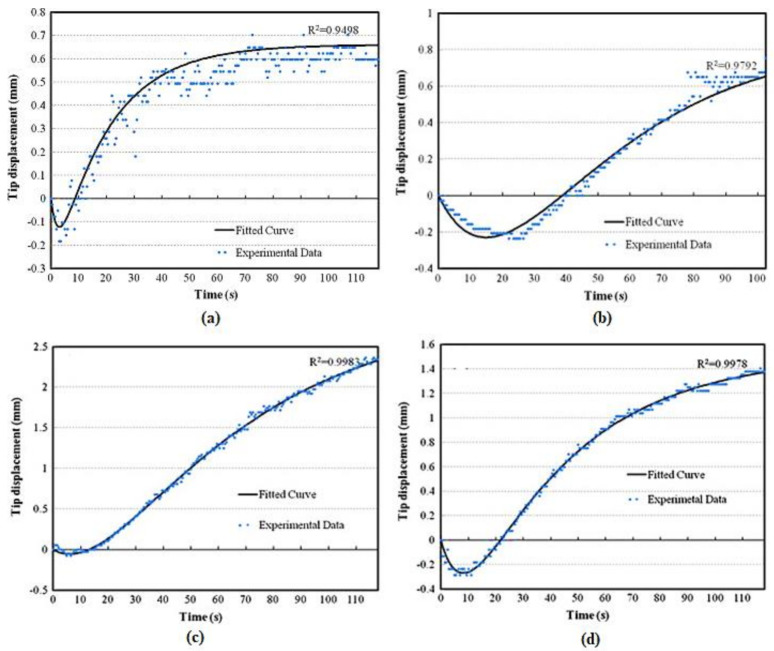
(**a**). Tip-displacement of the graphene-based polymer composite actuator in excitation-voltage of 3 V DC; (**b**). At 0.1 wt. percent graphene loading; (**c**). At 0.25 percent graphene loading and (**d**). At 0.5 percent graphene loading. Reproduced with permission from [108].

Recently, the electroactive performance of graphene-loaded cellulose composite actuators was reported by Sen et al. [108]. The films of microcrystalline cellulose (MCC) loaded with graphene nanoplatelets were prepared by the solvent casting method. An ionic liquid, 1-butyl-3-methylimidazolium chloride (BMCl), was used as the solvent. The composite films were converted into actuator strips by forming electrodes using gold leaf. The incorporation of graphene enhanced the conductivity and mechanical properties of the polymer composite. The actuator performance was measured at 3 to 7 V. The study reported that the graphene-based polymer composite loading reduced the actuation speed but operating at higher excitation voltages. Moreover, an increase of 267% in the maximum displacement at an excitation voltage of 3 V was achieved with the addition of graphene, as revealed from the Figure 11a–d. This clearly indicates that the loading of graphene nanoplatelets has led to improved electro-active actuator efficiency of the polymer composites.

Polymer nanocomposite based optical actuator is one of the fast-developing fields in contemporary research.

In a graphene–polymer composite actuator, the homogeneous dispersion of graphene is one of the important aspects to be ensured for good optical actuation behavior. The homogeneity of graphene dispersion was achieved by many researchers using functionalization techniques [109,110]. The IR triggered actuation in graphene–polydimethylsiloxane (PDMS) systems showed less photomechanical stress and strain. For example, 2 wt.% graphene nanoplatelet-PDMS system exhibited a change in stress of only less than 40 kPa [111]. Even with single layer graphene (1 wt.%), the photomechanical stress of the PDMS system was <50 kPa [112]. However, graphene-PDMS systems exhibited excellent reversibility.

Seema et al. reported a Thermally reduced graphene oxide (TRGO)-PDMS system with higher photomechanical stress of 133.44 kPa [113]. Even though the strain obtained was only 7.17%, the actuation behavior was 100% reversible.

Similarly, graphene/styrene-isoprene-styrene (SIS) copolymer composite was studied as an optical actuator [114]. The maximum photomechanical stress and strain obtained were 28.34 kPa and 3.1%, respectively. However, the on-off cycle of the actuator exhibited a marching behavior due to creep deformation, which became less prominent after a few on-off cycles.

Graphene–polymer composite actuators which can be driven by solvents, pH, chemicals, etc., can find a wide range of applications particularly in the biomedical field. A solvent driven actuator was developed by Deng et al. by pattering few-layer graphene (FLG) on an epoxy-based photoresist polymer (SU-8) [115]. The FLG/SU-8 bilayer can fold when immersed in water due to de-solvation of SU-8 or can unfold when immersed in acetone due to re-solvation of SU-8. Even after solvation and de-solvation, the graphene layer is held intact. This also puts up an opportunity to integrate graphene-based sensors on solvent driven self-folding polymer actuators.

### 3.6. Flexible Electronics

In recent years, there has been intensive research on flexible and stretchable electronics especially in the field of wearable electronic devices, stretchable circuits, flexible batteries, membrane keyboards, biomedical sensors, and artificial tissues. One of the critical parameters to realize flexible electronics is to retain the high conductivity under mechanical deformations.

**Figure 12:**
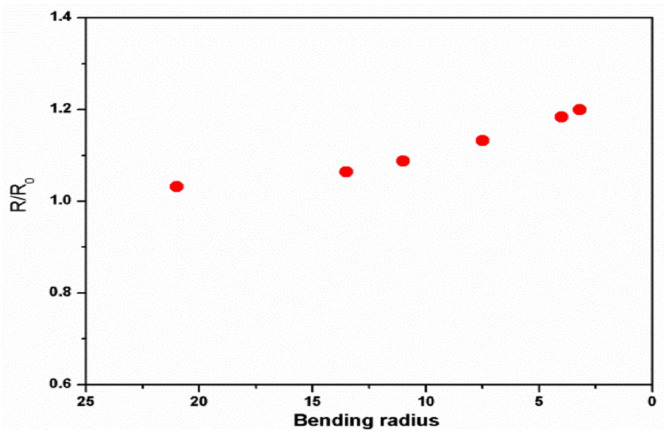
Variations in resistance as a function of bending-radius for the graphene aerogel/PDMS composites. Reproduced with permission from [116].

Wong et al. have developed a process to fabricate highly conductive and flexible graphene aerogel/PDMS composites. [116] A spontaneous reduction process prepared graphene aerogel, and due to its porous nature of this aerogel the incorporation of PDMS into the graphene framework was easier. Graphene aerogel/PDMS composite showed a high conductivity of 95 S/m at small filler loading of 0.8 wt%, as exhibited in Figure 12. The flexibility of the composites was studied by electromechanical tests and achieved good retention in conductivity (of about 80%) under various bending conditions.

### 3.7. Shape Memory Polymers

Incorporating conducting fillers into the shape memory polymer (SMP) is one of the most widely adopted methods to develop smarter shape memory materials. Many researchers have already succeeded in improving the shape memory effect by incorporating conducting fillers like graphene into the polymer matrix. In addition to imparting mechanical strength due to the high thermal conductivity of graphene, it can uniformly heat the SMP composites, which results in faster response and better shape recoverability.

A composite with triple stage shape memory performance was demonstrated by combining chemically modified graphene oxide with an interpenetrating polymer network of polyurethane with two different molecular weights [117].

**Figure 13:**
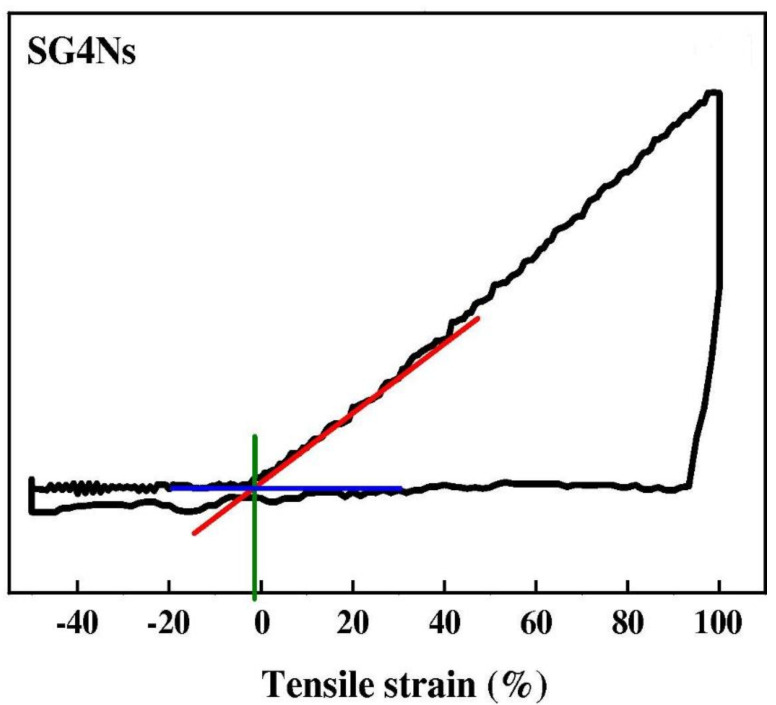
Thermal-shrinkage during physical-testing of pristine SMPU/GO nanofibrous mats. Reproduced with permission from [118].

**Figure 14:**
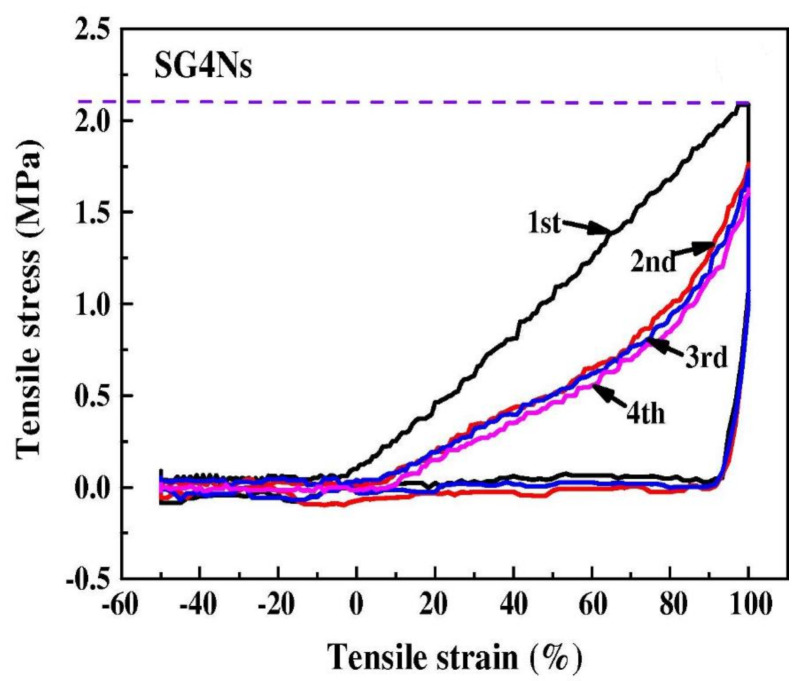
Average fixation-ratio of SMPU/GO nanofibrous mats during cyclic-tensile test resembling shape-memory effect. Reproduced with permission from [118].

The shape memory effect (SME) of graphene oxide (GO) incorporated shape memory polyurethane (SMPU) nanofibers were studied by thermal cyclic tests [118]. The SMPU/GO nanofibrous mats exhibited a better SME and lowered thermal shrinkage when compared with pristine SMPU nanofibrous mats. With a GO loading of 4.0 wt.%, a very low thermal shrinkage ratio of 4.7 ± 0.3%, a very high average fixation ratio, and a recovery ratio of 92.1% and 96.5% were obtained, exhibited from the Figure 13 and Figure 14, respectively [118]. The GO cross-linked SMPU molecular chains are not free to shrink and, hence, can be stabilized much more quickly for reaching the final structure, which results in high fixation and recovery ratio.

In a similar study, the correlation between the temperature dependent SME, and the cross-linking density in GO/polyurethane nano composites was established by Ponnamma et al. [119]. As the filler concentration was varied, the crosslinking density was also varied affecting the shape memory properties.

Graphene–polymer composites have emerged as one of the most promising materials that can revolutionize the field of electronics and optoelectronics.

### 3.8. Optical Limiting Applications

Optical limiting is a mechanism by which certain materials, which are transparent to light at low intensities, restrict the transmission of light above a threshold input intensity. Graphene, GO, and RGO exhibited high nonlinear optical absorption, which makes them suitable as good optical limiters. Most of the studies were based on dispersions in liquid. However, for practical device applications, these need to be used as solid materials, and the dispersion of graphene or GO or RGO in the solid matrix is very important. There have been a few studies on the nonlinear optical properties of RGO in various solid matrices including glass and polymers. The nonlinear optical properties of covalently functionalized GO in silica gel glasses were studied by Tao et al. [120]. They have observed that the nonlinear optical response of functionalized GO was better in silica gel glass than in deionized water.

Similarly, RGO with porphyrin incorporated in polymers was studied as optical limiters by a few researchers [121]. There have been a few reports on the optical limiting properties of graphene–polymer composites also. However, in order to avoid the dispersibility issues of graphene, functionalization was required in most of the cases [122,123].

### 3.9. Biomedical Applications

Conducting polymers have innumerable applications in the field of medical science like drug delivery, biomedical implants, tissue engineering, and diabetic monitoring.

#### 3.9.1. Drug Delivery

During last few years conducting polymers have been used in drug delivery due to biomedical compatibility. These are good for drug release applications [124]. The choice of drug delivery method depends upon the types of drug and types of treatment required. The routes of drug delivery are peroral, gastrointestinal, rectal, ocular, intravaginal, transdermal, vascular injection, nasal, and pulmonary [125]. The different types of drugs like anionic, cationic or neutral can be injected into the polymer backbone [126].

The surface area of conducting polymer films can be increased by titanium and carbon nanotubes to store drugs [127,128]. Many therapeutic drugs, such as 2-ethylhexyl phosphate, dopamine, naproxen, heparin, and dexamethasone, have been bound and successfully released from these polymers [129,130,131,132,133,134,135,136,137,138,139,140,141,142,143,144].

#### 3.9.2. Tissue Engineering

Conducting polymers also find applications in tissue engineering due to their stimulus-responsive property. The composites of these polymers act as substrates that promote cell growth, adhesion, and proliferation at the polymer-tissue interface [130,131,145,146,147,148,149,150,151,152,153,154,155,156,157,158].

#### 3.9.3. Diabetic Monitoring

Conducting polymers and their nanocomposites have advantages in the diagnosis and treatment of diabetes. These are used for the manufacturing of devices that are needed in the diabetes treatment of human beings. The pros of employing conducting polymers in diabetic-treatment are that their physical and chemical characteristics are being modified by doping with numerous chemical agents whenever required. The glucose biosensor is used in the treatment of diabetes. Conducting polymers are applied in closed loop delivery devices, which are needed for diabetic patients [132].

Thus, conducting polymers containing metal particles possess interesting properties of scientific and practical interests [132,133,134,135,136,137,138,139,140,141,142,143,144,145,146,147,148]. Researchers are more interested in developing the three-dimensional structure of conducting polymers, hybrid, and nanohybrid materials of conducting polymers. The hybrid and nanohybrid conducting polymers are synthesized by adding metal, metal oxides, graphene, and graphene oxide to conducting polymers. These new materials improve functionality in different areas like sensors, electronic devices, and biomedical application. The graphene nanohybrid of these polymers is used as an electrode in synthesis of capacitors. These nanohybrid materials increase stability, flexibility, and capacity of capacitors [149,150,151,152,153,154,155,156,157,158,159,160,161]. The different properties of polymers like thermal stability, mechanical properties, conductivity, corrosion protection properties on steel and aluminium can be escalated to a firm extent by doping.

## 4. Novel Polymer Nanocomposite Materials for Multifunctional Engineering Applications

Li et al. outline the synthetic–polymeric composite biomedical-coatings comprising of inorganic elements, but also present their design methodologies and manufacturing processes. For developing composites coatings, synthetic as well as polymeric elements can indeed be blended prior to applying coating methods, yet they can be subsequently coated and sprayed onto surfaces. Generally, the operations really are not challenging [162].

For most scenarios, polymerics serve as hosting matrixes while inorganic particles act as dispersing filling materials, emphasizing the existing concern that elements of composite coatings generally exhibit their merits and take measures in such a discrete way instead of in a spontaneous synergistic manner. Additionally, study on intermolecular-level interfacial contact and interactions among synthetic and polymeric elements is inadequate [162].

How else to optimize the proportion and focus on improving functionality and suitability of the elements, as well as how to strengthen the stimulatory synergic effect to develop novel bio-functions, might just be the upcoming challenging issue for polymeric composite coatings, which also relies heavily on a comprehensive understanding of the interactions among polymeric materials as well as inorganic particles. It is indeed worthwhile to examine alternative prospects for synthetic–polymeric composite coatings, including their realistic medical applications [162].

Rikhari et al. mixed graphene–oxide (GO) with a conducting poly-pyrrole (PPy) polymeric and then deposited it on titanium material, employing a traditional electropolymerization process. Such hybrid coatings exhibited excellent resistance to corrosion, and bio-compatibility, rendering them attractive for orthopaedic implants, bone-regeneration, and biomedical applications [163].

**Figure 15:**
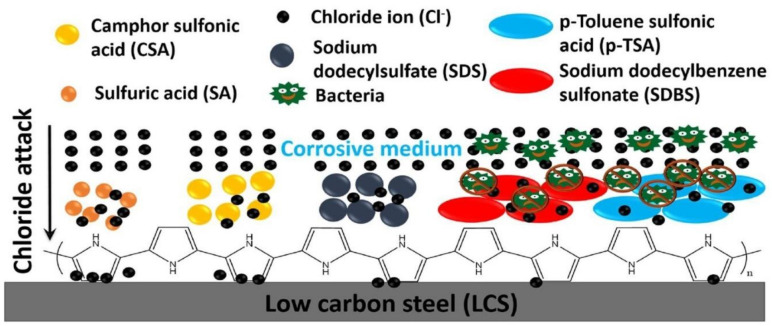
Poly-pyrrole (PPy) polymeric-coatings on carbon-steel for bio-medical applications. Reproduced with permission from [164].

Sulfonic acids were being utilized as doping agents in the electro-chemical synthesis of poly-pyrrole (PPy) coatings on carbon-steel as reported by the Nautiyal et al. [164]. The influence of acidity doping (p-toluene sulfonic-acid (p-T.S.A.), sulfuric-acid (S.A.), camphor-sulfonic-acid (C.S.A.)) upon carbon-steel surface modification passivation was analyzed employing linear-potentio-dynamics and contrasted to a coating’s microstructure as well as corrosive shielding effectiveness as showed in Figure 15. The sorts of doping agents utilized had a substantial influence on the coating’s potential to shield the surface of the metal against chloride-ion attacks. The corrosive behavior of a polymeric backbone is primarily determined by the volume, structure, magnitude, size, positioning, and orientation of the doping agent. Furthermore, both p-T.S.A. and sodium-do-decyl-benzene-sulfonate (S.D.B.S.) have included additional benzene-rings, which stacked around each other to produce lamellar-membranous sheet-like barriers to chloride-ions, rendering them effective doping agents for PPy coatings for reducing corrosion to a considerable extent. Additionally, adhesive ability was strengthened via directly introducing long-chain carboxylic-acid into to the monomer’s solvent. Besides that, PPy coatings coated with SDBS indicated remarkable bio-cidal characteristics against Staphylococci. PPy coatings on carbon-steels with double anticorrosion and superior bio-cidal abilities have such a promising possibility to be used throughout the industries for anticorrosion and anti-microbial applications [164].

The phenomena of static-charge are unforeseeable, especially when an airplane is traveling at higher latitudes, inflicting the accumulation of static charge-carriers to surpass a specified threshold, resulting in the breakdown of its components and subsystems, involving devastating explosive blasts and radio-transmission malfunction loss, as revealed by Yadav et al. [165]. The accumulating of static charge-carriers on aeroplanes is caused by the interaction among the aircraft’s outermost exterior surface-layer as well as the external atmospheric characteristics, which would include surrounding air, snow, hailstorm, dirt, and volcanism eruption-ash, in addition to its tribo-electric charging. Because of the lightweight materials, and similar physicomechanical characteristics, innovative polymeric-based composites or nanocomposites have become prevalent structural constituents for aviation sectors throughout the last few decades. However, such polymeric composites do not provide low-resistance trajectory for electric charge transport, thus, attempting to make them susceptible to influence of thunderstorms, lightning-attacks, and p-static interference. For this perspective, nano-filler formulations are critical for developing conducting polymeric composite structures using nonconductive polymeric matrices. With the emergence of carbon-derived polymeric nanocomposites, certain challenges pertaining to non-conductive polymeric matrices have indeed been satisfactorily resolved, and the composites have evolved into an avantgarde genre [165].

Owing to its remarkable electrical properties, redox characteristic, but also functionality at ambient temperatures, novel nanocomposites appertaining to graphene and conducting polymerics like poly-aniline (P.A.N.i.), poly-pyrrole (PPy), poly (3,4 ethyl-dioxy-thiophene) (P.E.D.O.T.), poly-thiophene (PTh), as well as their compound-derivatives have notably become extensively utilized as active materials throughout gas-sensing applications as enumerated by the Zamiri et al. [166] Whenever these two materials have been blended, they excelled, pure graphene as well as conducting polymerics, in terms of sensors-based characteristics. This could be attributable to the nanocomposites’ high specific surface area, and indeed the combined synergic effects of graphene and conductive polymerics. A kind of graphene, as well as conductive polymeric nanocomposites processing techniques, including in situ polymerization, electro-polymerization, solution-blending, selfassemblage, and many others, have been revealed, and utilization of such nanocomposites as sensing materials has also been shown to enhance the effectiveness of gas-sensing.

A kind of graphene, as well as conductive polymeric nano-composites processing techniques, including in situ polymerization, electro-polymerization, solution-blending, selfassemblage, and many others, have been revealed, and utilisation of such nanocomposites as sensing materials has also been shown to enhance the effectiveness of gas-sensing [166].

**Figure 16:**
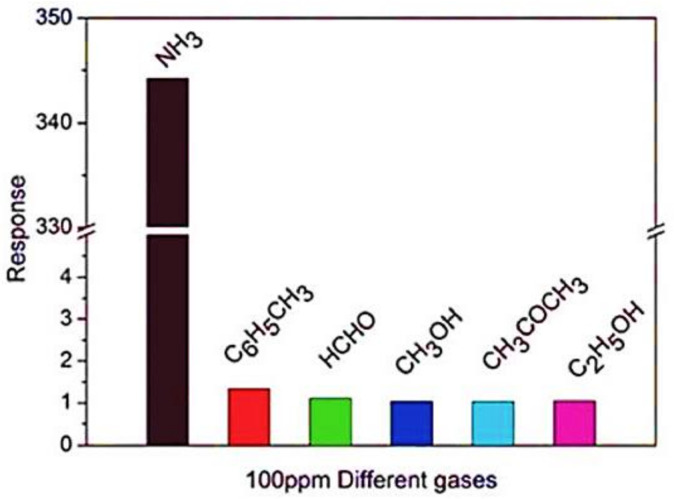
Sensitivity response of reduced graphene-oxide–PANI hybridized thin films towards various gases at 100 ppm. Reproduced with permission from [166].

Findings unveiled that the reduced graphene oxide–P.A.N.I. blends packed on a flexible-based poly-ethylene-terephthalates thin film demonstrated the maximum response of 344.12 to 100 ppm ammonia, the gas sensor activity of P.E.D.O.T./reduced graphene oxide nanocomposites processed through in situ-polymerization method unveiled outstanding sensing effectiveness to nitrogen-dioxide, and reduced graphene oxide/PPy nanocomposite demonstrated the maximum sensitivity of 102 percent under 50 ppm as displayed in Figure 16 [166].

Conducting-polymerics with ultra-high conductivity, and electro-chemical characteristics have attracted the attention of investigators as catalysis accelerators for polymeric-electrolytic-membrane-based fuel-cells (P.E.M.F.C.’s) including microbial fuel-cells (M.F.C.’s) as discussed by Ghosh et al. [167]. Furthermore, metallic or metallic-oxide catalytic-accelerators can be immobilized on the surface of a virgin polymer, or a biocompatible-polymer to produce conductive polymeric-based nano-hybrids (C.P.N.H.’s) having excellent catalyzed activity and durability. Transition metal-oxides, which provide greater superficial surface area and porosity and permeable nanostructures, exhibited distinctive synergic activity with conducting polymerics. As a result, C.P.N.H.’s could be employed to produce a stabilized, environment-friendly bio- or electrocatalyst, exhibiting enhanced catalytic performance as well as an increased electron charge particle transfer rate. Palladium/poly-pyrrole (PPy) C.P.N.H.’s possesses 7.5 and 78 times more mass-activities of commercialized palladium/carbon, and bulk palladium/PPy used as anode materials for ethanol-oxidation, respectively. The electro-catalytic activities of Palladium-rich multi-metallic alloy-compositions placed on PPy nanofibrous was roughly 5.5 times more than the mono-metallic counter-parts. Likewise, binary and ternary platinum-rich electro-catalysts displayed higher catalysis performance for the methyl-alcohol-oxidation, as well as the catalysis behavior of Pt-24Pd-26Au-50/PPy considerably enhanced up to 12.45 A/mg platinum, which itself is around fifteen times superior than those of commercialized platinum/carbon (0.85 A/mg platinum) [167].

Outcomes also reported that such a novel class of C.P.s-based bio-materials can still be employed in cellular investigations, like tissue-engineered, regenerative-medicine, bioengineering, bio-medical equipment, scaffoldings, and so on. It might be immensely favorable to the progress of such a domain [168].

## 5. Drawbacks of Conducting-Polymers

Conducting-polymerics (C.P.’s) with multifunctional-applications have had some constraints owing to their toxic-carcinogenic character and disparities in in vivo and in vitro investigations. In general, the crucial challenges for C.P.’s include physical characteristics, cytocompatibility, bioactivity, and bio-compatibility.

Though C.P.’s have tremendous promising opportunities in multi-functional applications, they have numerous downsides due to the initial bursting drug-delivery release-rate, and the hydrophobic characteristics of the polymeric, thus, hindering their usage. Nonetheless, drug-releasing mechanisms seem to be of enormous interest to scientists as well as providing possibilities in treating cancer, and in minimal invasive-methods for myriads of neuro, and cardio-vascular treatments.

Another key drawback of existing C.P.’s are as follows: the cyclic-stabilisation is worse than that of carbon materials, and the energy-capacity, power-density, specific-capacitive, and specific-power are significantly low while comparing to transition metal-oxides.

## 6. Concluding Remarks and Future-Outlook

Nowadays, great advancements in conducting polymers and rare earth oxides have been achieved in electrochemical and conductors by modifying the surface of the working electrode. This review paper gives sound information about the chemical and electrochemical synthesis methods and applications of conducting polymers in different fields, like electronic devices, sensors, protection of corrosion, shape memory polymers, actuators, flexible electronics, optical limiting, drug delivery, and tissue engineering. The nanocomposites and nanohybrid materials of conducting polymers improve the useful properties of polymers in different fields. An improved thermal and cyclic stability with a low internal resistance of the composites was observed with the application as dielectric, antistatic properties, semiconductor, and energy storage devices. Biopolymeric nanocomposites for storing energy, power-generation, energy production, conservation of energy, and corrosion inhibitors purposes have indeed been presented in this article. ICP nanocomposites have piqued the curiosity of researchers due to their high electrical properties, being relatively inexpensive, the ease of manufacture, and their biodegradability. Graphene and certain other nano-fillers are proliferating and gaining momentum as appealing possibilities for nanocomposites. Progressions in the fabrication, evaluation, testing, characterization, and computation modeling of nanostructures have offered ample and endless possibilities for tailoring the engineering characteristics of PNC frameworks.

Corrosive inhibitors and anodic shielding are the two foremost significant methods for reducing the metal corrosion rates. Composite-conducting polymerics (C.C.P.’s) were shown to offer excellent corrosion-resistant characteristics compared to conductive polymeric-coating materials. This seems to be attributable to the nanoadditive’s increased area of surface for dopant-releasing and the development of a barriers impact versus diffusion transport. It is predicted that in upcoming investigations, a plethora of reinforcing particulates will be at the forefront of interest, with a heavy emphasis on the application of C.C.P.’s on certain metal surfaces and in diverse domains. Furthermore, since corrosive resistance by C.P.’s is predominantly focused on anodic shielding, the stabilization of the passive oxide layer underneath the polymeric coating and the inhibitors of aggressive anion-ions from penetrating the polymeric membrane must, therefore, be thoroughly studied.

The toxic effect of electro-active poly(aniline)-based oligomers has indeed been thoroughly examined, and it is a restrictive concern for bio-medical applications. Throughout this domain, suggestions for novel electro-active oligomers predicated on better stabilized, and bio-compatible electro-active monomeric-subunits and/or oligomers could be of interest. A further approach would be to utilize a relatively minimal, aniline-based oligomer concentration, or even to explore newer possibilities to escalate bio-compatibility.

Electro-active macro-monomers appear as a promising strategy for obtaining grafting co-polymers with conductance, and bio-degradability. Substantial research is required, nevertheless, before these bio-materials can still be employed in cellular investigations, like tissue-engineered, regenerative-medicine, bioengineering, bio-medical equipment, scaffoldings, and so on. It might be immensely favorable to the progress of such a domain.

Some other crucial trend is that it would be worthwhile not only to synthesize novel materials, but also to delve deeply into their characterization. Significant investigations have recently concentrated on the bio-interface of materials with cellular, either by investigating nano-sized scaling characteristics or interacting with bio-molecules, in order to properly comprehend what could be better for cellular interface interaction. Subsequently, the insights could contribute to developing intelligent bio-materials inside the upcoming years.

C.P.’s are generally infusible, and unsolvable, rendering treatment of such materials challenging, particularly when mixed with the other polymeric matrices for anti-static applications. Even so, with advancement of manufacturing methods, the afore-mentioned concern is now being overcome, and C.P.’s are showing promise in conductive materials in anti-static applications, owing to their convenience of synthesizing, their lower-density, their light-color, and the manageable electrical characteristics, between whereby the water-based conductive coatings have been remarkably alluring, deserving additional endeavour for useful anti-static devices.

Nevertheless, numerous impediments have yet to be overcome. The first one is the paucity of elevated nano-fillers, as well as the exorbitant operating costs and complexity in scaling-up. The next difficulty is correlated with the handling and treatment of PNCs. Controlling a dispersal scattering and orientations of the nano-fillers is paramount for optimising the functionality efficacy of PNCs. The final barrier is a dearth of comprehensive insight and prediction capabilities of the recurring viable fundamental processing structural–characteristic interactions necessary to completely maximize the commercialization prospects of such innovative biomaterials. The generated models frequently failed to anticipate scientific findings. Furthermore, substantial extensive research attempts to generate breakthrough innovative biopolymer nano-composites employing cleaner sustainable, economical, environmentally-sound, and alternative renewable sources of energy are desired from the standpoint perspective of attaining sustainable development.

## Figures and Tables

**Table 1:** Applications of various rare earth oxides based conducting polymer nanocomposites.

Polymer	REO	Applications	References
Poly(ethylene oxide) (PEO)	La_2_O_3_	Semiconductor and Solid Polymer Electrolyte (SPE)	[4]
Polyaniline (PANI)	La0.67Sr0.33MnO_3_	Sensor	[110]
PANI	Sm_2_O_3_, La_2_O_3_	Thermally stable material	[111]
PANI	CeO_2_	Thermally stable material	[112]
PANI	CeO_2_	Semiconductor and supercapacitor	[112]
PANI	La-Nd	Electromagnetic Interference	[11]
PANI	Ce-TiO_2_	Sensor	[113]
PANI	CeO_2_, Dy_2_O_3_	Thermally stable material	[114]
PANI	Terbium(iii)	Light Emitting Diode	[10]
PANI	WO_3_	Sensing	[115]
PANI	Nd_2_O_3_:Al_2_O_3_	Dielectric constant	[116]
Polycarbazole	-	Semiconductor	[117]
Polyindole (PIN)	TiO_2_	Semiconductor	[118]
PIN	Y_2_O_3_	Dielectric constant	[119]
Polypyrrole (PPY)	CeO_2_	Semiconductor	[120]
PPY	CeO_2_	Sensor	[121]
PPY	Nb_2_O_5_	Semiconductor	[122]
PPY	Y_2_O_3_	Semiconductor	[2]
PPY	Sm_2_O_3_	Supercapacitor	[6]
PPY	Y_2_O_3_	Batteries, sensors and actuators	[123]
PPY	La^3+^, Sm^3+^, Tb^3+^, Eu^3+^	Supercapacitor	[124]
PPY	RuO_2_	Supercapacitor	[9]
PPY	Eu_2_O_3_	Supercapacitor	[125]
PPY	Y_2_O_3_	Dielectric constant	[123]
Polyvinyl Alcohol (PVA)	Ho^3+^, Gd^3+^	Optical display	[126]
PVA/PPY	-	Dielectric	[127]
Polyvinylidene fluoride (PVDF)	La_2_O_3_	Thermally stable material	[128]

**Table 2:** Comparison of Chemical and Electrochemical Polymerization.

Chemical Polymerization	Electrochemical Polymerization
Yield of the product is large in amount	Yield is less, and synthesis of the thin film is possible
Synthesis is difficult	Synthesis is quite easy
They do not offer control of polymerization and doping level	In this method, polymerization and doping levels can be controlled
Doping and polymerization do not occur simultaneously	Doping and polymerization occur simultaneously
Polymer is easily collected and packed	Difficult to remove the film from the electrode surface

## Data Availability

No data were used to support this study.
